# Long noncoding RNA PCAT-14 induces proliferation and invasion by hepatocellular carcinoma cells by inducing methylation of miR-372

**DOI:** 10.18632/oncotarget.16260

**Published:** 2017-03-16

**Authors:** Yawei Wang, Ye Hu, Gang Wu, Ye Yang, Yanqing Tang, Wanchuan Zhang, Kaiyu Wang, Yu Liu, Xin Wang, Tiemin Li

**Affiliations:** ^1^ Department of Geriatric Surgery, The First Affiliated Hospital of China Medical University, Shenyang, Liaoning 110001, China; ^2^ Department of Nephrology, Liaoning Provincial People's Hospital, Shenyang, Liaoning 110000, China; ^3^ Department of General Surgery, The First Hospital Affiliated to China Medical University, Shenyang, Liaoning 110001, China; ^4^ Department of Psychology, The First Affiliated Hospital of China Medical University, Shenyang, Liaoning 110001, China

**Keywords:** PCAT-14, miR-372, HCC, proliferation, invasion

## Abstract

Long non-coding RNAs (lncRNAs) regulate oncogenesis by inducing methylation of CpG islands to silence target genes. Here we show that the lncRNA PCAT-14 is overexpressed in patients with hepatocellular carcinoma (HCC), and is associated with a poor prognosis after surgery. Our results demonstrate that PCAT-14 promotes proliferation, invasion, and cell cycle arrest in HCC cells. In addition, PCAT-14 inhibits miR-372 expression by inducing methylation of the miR-372 promoter. Simultaneously, miR-372 eliminates the effects of PCAT-14 on proliferation, invasion, and cell cycle in HCC cells. Moreover, PCAT-14 regulates expression of ATAD2 and activation of the Hedgehog pathway via miR-372. These findings indicate that PCAT-14 plays an important role in HCC, and may serve as a novel prognostic factor and therapeutic target.

## INTRODUCTION

Hepatocellular carcinoma (HCC) is one of the most prevalent tumor types with the highest mortality rate [1]. The occurrence of HCC is increasing, especially in Asia and Africa [2]. Many HCC patients do not have any symptoms until an advanced stage [3]. Even though HCC prognosis has improved thanks to the development of effective surgical techniques and diagnostic methods over recent years, long-term prognosis is still unsatisfactory largely due to the high recurrence (50–70% at 5 years) [4, 5]. Thus, there is an urgent need to identify valuable diagnostic and prognostic biomarkers to improve clinical outcomes and develop effective individual therapeutic strategies for patients with HCC.

Long non-coding RNAs (lncRNAs) are a class of non-coding RNAs that have more than 200 nucleotides (nt) [6, 7]. With the development of deep sequencing technologies, lncRNAs have increasingly been associated with various human diseases, particularly different types of cancer [8–10]. LncRNAs, such as HULC, HOTAIR, and DBH-AS1 represent an emerging group, which may regulate HCC cell proliferation, migration, and apoptosis [11–13].

LncRNA prostate cancer-associated transcripts (PCATs) were originally identified as biomarkers for prostate cancer [14]. They include PCAT-1, PCAT-6, PCAT-14, and PCAT-29, which play critical roles in the progression of several cancer types [15–17]. Increased expression of PCAT-1 has been associated with advanced clinical parameters and poor overall survival of HCC patients [18]. Upregulation of PCAT-1 contributes to HCC cell proliferation, migration, and apoptosis [19]. PCAT-14 is located on chromosome 22, GRCh38.p7. An integrative analysis revealed that PCAT-14 is the most prevalent lncRNA that is aberrantly expressed in prostate cancer patients [20], and predicts a poor outcome [21]. However, the expression and function of PCAT-14 in HCC have not been studied.

In this study, we have analyzed the expression and function of PCAT-14 in HCC. Our results demonstrate that the PCAT-14 expression is associated with HCC metastasis, tumor size, and TNM stage, suggesting that PCAT-14 may be involved in the tumorigenesis and progression of HCC. In addition, Kaplan–Meier and Cox regression analysis shows that high PCAT-14 expression correlates with poor OS, indicating that it could serve as an independent prognostic factor for overall survival in HCC. Our results demonstrate that PCAT-14 promotes cellular invasion and proliferation, and inhibits miR-372 expression by inducing methylation of CpG islands in the miR-372 promoter. In addition, PCAT-14 regulates ATAD2 expression and activation of Hedgehog pathway in HCC cells, depending on miR-372. Together, these results indicate that PCAT-14 plays a critical role in HCC, and may serve as a candidate target for new HCC therapies.

## RESULTS

### PCAT-14 expression is increased in HCC tissues and cells

**Figure 1:**
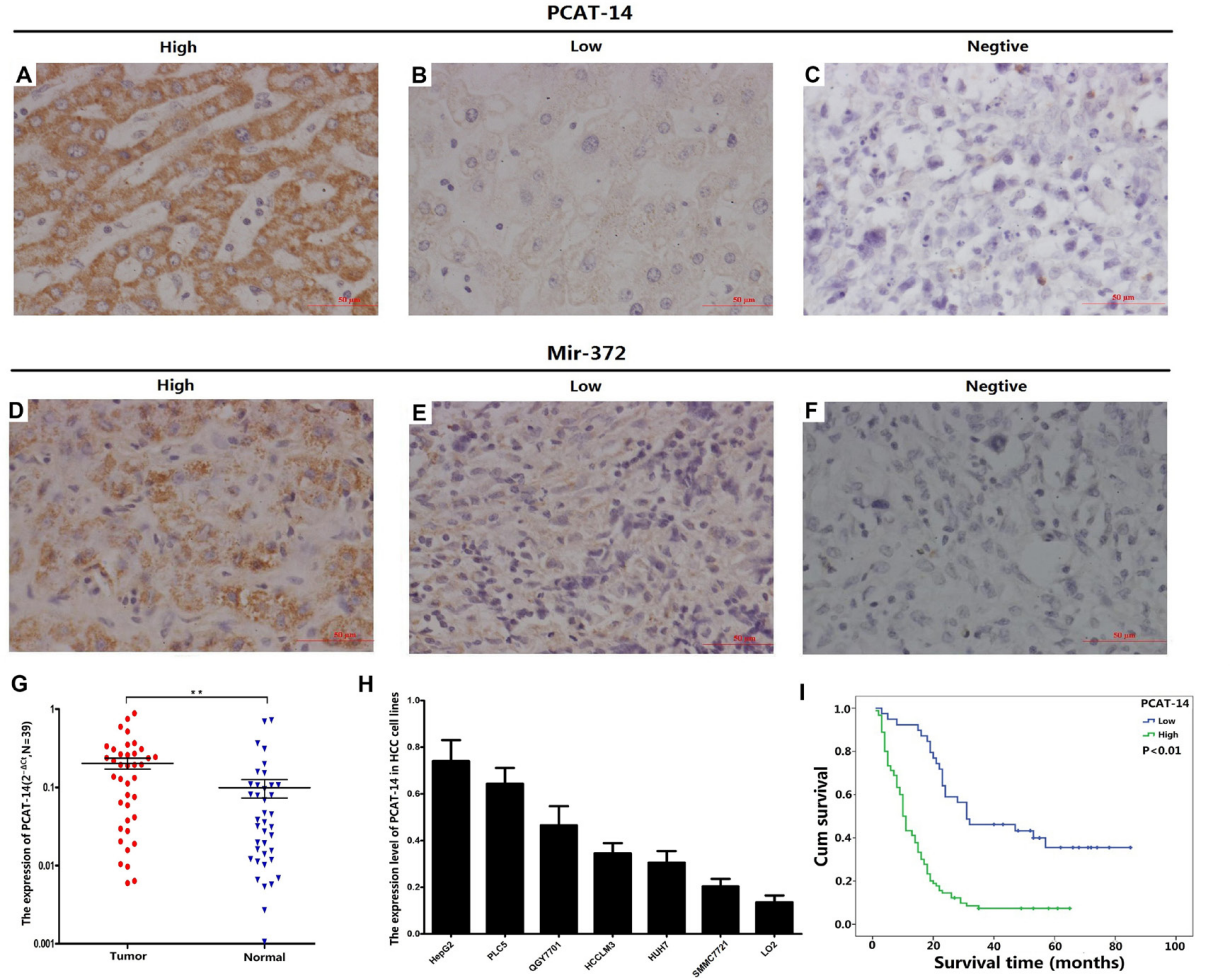
**(A–C)** The expression of PCAT-14 by *in situ* hybridization; (**D**–**F**) The expression of miR-372 by *in situ* hybridization; (**G**) The expression of PCAT-14 by qRT-PCR in 39 HCC patients (*P* < 0.01); (**H**) The expression of PCAT-14 in HCC cell lines; (**I**) Overall survival of HCC patients in relation to PCAT-14 expression according to *in situ* hybridization in 120 HCC patients. Survival of HCC patients with high PCAT-14 expression versus low expression (*P* < 0.01).

RT-PCR analysis demonstrated an increased PCAT-14 expression in HCC tissues compared with normal tissues (0.203 ± 0.204 vs. 0.099 ± 0.164, respectively; *P* < 0.01) (Figure 1G). *In situ* hybridization of tissues from 120 HCC patients revealed that the PCAT-14 expression was increased in 91 cases (75.8%) (Figure 1A–1C). Furthermore, liver LO2 cells had decreased PCAT-14 expression compared to HCC cell lines HepG2, PLC5, QGY7701, HCCLM3, HUH7, and SMMC7721 (Figure 1H).

### Clinical significance of PCAT-14 expression in HCC

According to the results of *in situ* hybridization, the association between PCAT-14 expression and clinicopathological factors of the 120 HCC patients was analyzed. The expression of PCAT-14 was significantly higher in HCC tissues with advanced TNM stage compared with those with early TNM stage (*P* = 0.021). In addition, the increased PCAT-14 expression was associated with tumor metastasis (*P* = 0.022) and larger tumor size (*P* = 0.006, Table 1). The OS was higher in HCC patients with lower PCAT-14 expression than in those with higher PCAT-14 expression (*P* < 0.01, Figure 1I). In addition, a multivariate analysis using the Cox model indicated that PCAT-14 expression, metastasis, and AFP status were independent, poor prognostic factors (Table 2).

**Table 1:** Association between PCAT-14 expression according to *in situ* hybridization and conventional clinicopathological parameters in 120 patients with HCC

Characteristics	Number of patients	PCAT-14 Lowexpression	PCAT-14 Highexpression	*P*
	120	29	91	
Age (years)				
≥ 50	74	14(18.9%)	60 (81.1%)	0.089
< 50	46	15 (32.6%)	31 (67.4%)	
Gender				
Male	51	16 (31.4%)	35 (68.6%)	0.113
Female	69	13 (18.8%)	56 (81.2%)	
Tumor size				
≥ 5cm	51	6 (11.8%)	45 (88.2%)	**0.006**
< 5cm	69	23 (33.3%)	46 (66.7%)	
Metastasis				
Yes	37	4 (10.8%)	33 (89.2%)	**0.022**
No	83	25 (30.1%)	58 (69.1%)	
HBsAg status				
Positive	72	19 (26.4%)	53 (73.6%)	0.486
Negative	48	10 (20.8%)	38 (79.2%)	
Tumor differentiation				
High	43	12 (27.9%)	31 (72.1%)	0.715
Moderate	44	9 (20.5%)	35 (79.5%)	
Poor	33	8 (24.2%)	25 (75.8%)	
Cirrhosis				
Yes	73	17 (23.3%)	56 (76.7%)	0.781
No	47	12 (25.5%)	35 (74.5%)	
Serum AFP				
< 200 ng/dl	68	20 (29.4%)	48 (70.6%)	0.106
≥ 200 ng/dl	52	9 (17.3%)	43 (82.7%)	
Recurrence*				
Yes	34	8 (23.5%)	26 (76.5%)	0.918
No	86	21 (24.4%)	65 (75.6%)	
TNM stage				
I	18	7 (38.9%)	11 (61.1%)	**0.021**
II	32	12 (37.5%)	20 (62.5%)	
II	40	6 (15%)	34 (85%)	
IV	30	4 (13.3%)	26 (86.6%)	

Notes: Bold fonts indicate Statistically significant. Recurrence*:follow-up time for five years after therapeutic surgery.

**Table 2:** COX regeression regression analysis on the relationship of clinicopathologic characteristics and prognosis

Characteristics	Univariate			Multivariate		
	HR	CI(95%)	*P*	HR	CI(95%)	*P*
PCAT-14	1.811	1.321–2.403	0.009	1.765	1.110-2.736	0.012
Age	0.982	0.671–1.437	0.924			
Gender	1.113	0.729–1.698	0.620			
Tumor stage	1.098	0.810–1.488	0.547			
Tumor differentiation	1.080	0.800–1.456	0.616			
Metastasis	1.942	1.319–2.861	0.001	1.711	1.098–2.654	0.011
Tumor size	1.334	0.896–1.986	0.156			
Serum AFP	2.280	1.482–3.505	0.0001	1.983	1.245–3.092	0.007
Cirrhosis	1.310	0.894–1.921	0.166			

Notes: Bold fonts indicate Statistically significant.

### PCAT-14 induces cancer cell invasion

**Figure 2:**
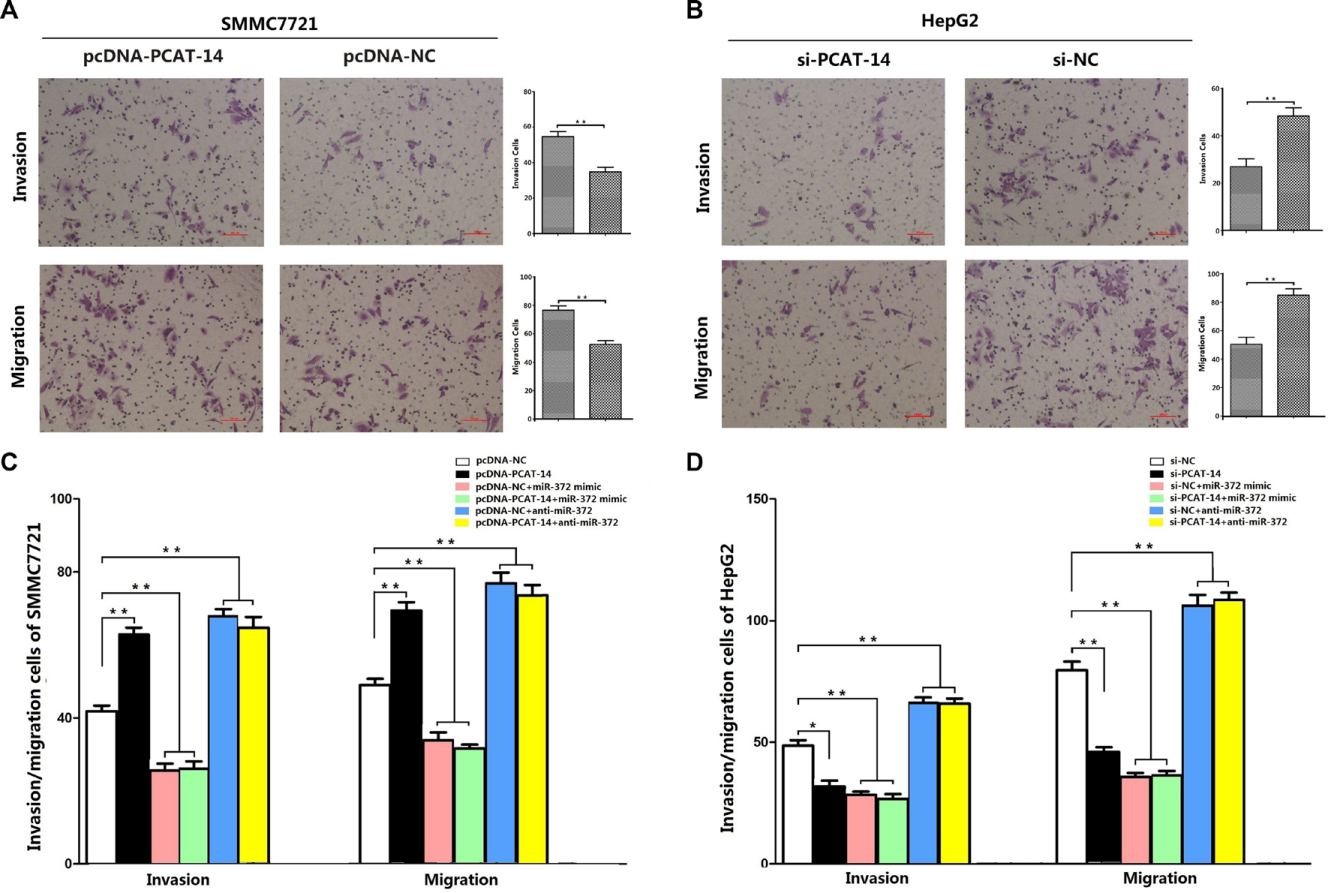
(A) Transwell assays of SMMC7721 cells transfected with pcDNA-PCAT-14 and pcDNA-NC:PCAT-14 up-regulation had a acceleration effect on cell invasion and migration in SMMC7721 cells (*P* < 0.01); (**B**) Transwell assays of HepG2 cells transfected with si-PCAT-14 and si-NC:PCAT-14 konckdown had a measurable inhibitory effect on cell invasion and migration in HepG2 cells (*P* < 0.01); (**C**) Transwell assays of SMMC7721 cells transfected with pcDNA-NC, pcDNA-PCAT-14, pcDNA-NC+miR-372 mimic, pcDNA-PCAT-14+miR-372 mimic, pcDNA-NC+anti-miR-372, pcDNA-PCAT-14+anti-miR-372:miR-372 could eliminate the effect of PCAT-14 overexpression on invasion and migration in SMMC7721 cells (*P* < 0.01); (**D**) Transwell assays of HepG2 cells transfected with si-NC, si-PCAT-14, si-NC+miR-372 mimic, si-PCAT-14+miR-372 mimic, si-NC+anti-miR-372, si-PCAT-14+anti-miR-372:miR-372 could eliminate the effect of PCAT-14 knockdown on invasion and migration in HepG2 cells (*P* < 0.01).

Cell invasion and migration assays demonstrated that SMMC7721 liver cancer cells transfected with pcDNA-lincRNA-PCAT-14 displayed more invasive and migratory properties compared to cells transfected with pcDNA-NC (invasion cells: 55 ± 8 VS. 35 ± 6, *P* < 0.01; migration cells: 77 ± 9 vs 53 ± 6, *P* < 0.01, Figure 2A). Trans-well chamber assay also showed that downregulation of PCAT-14 by transfected si-lincRNA-PCAT-14 in HepG2 cells significantly inhibited their invasion and migration compared with si-NC groups (invasion cells: 27 ± 9 VS. 49 ± 11, *P* < 0.01;migration cells: 51 ± 12 vs 79 ± 10, *P* < 0.01, Figure 2B).

### PCAT-14 induces cancer cell proliferation

**Figure 3:**
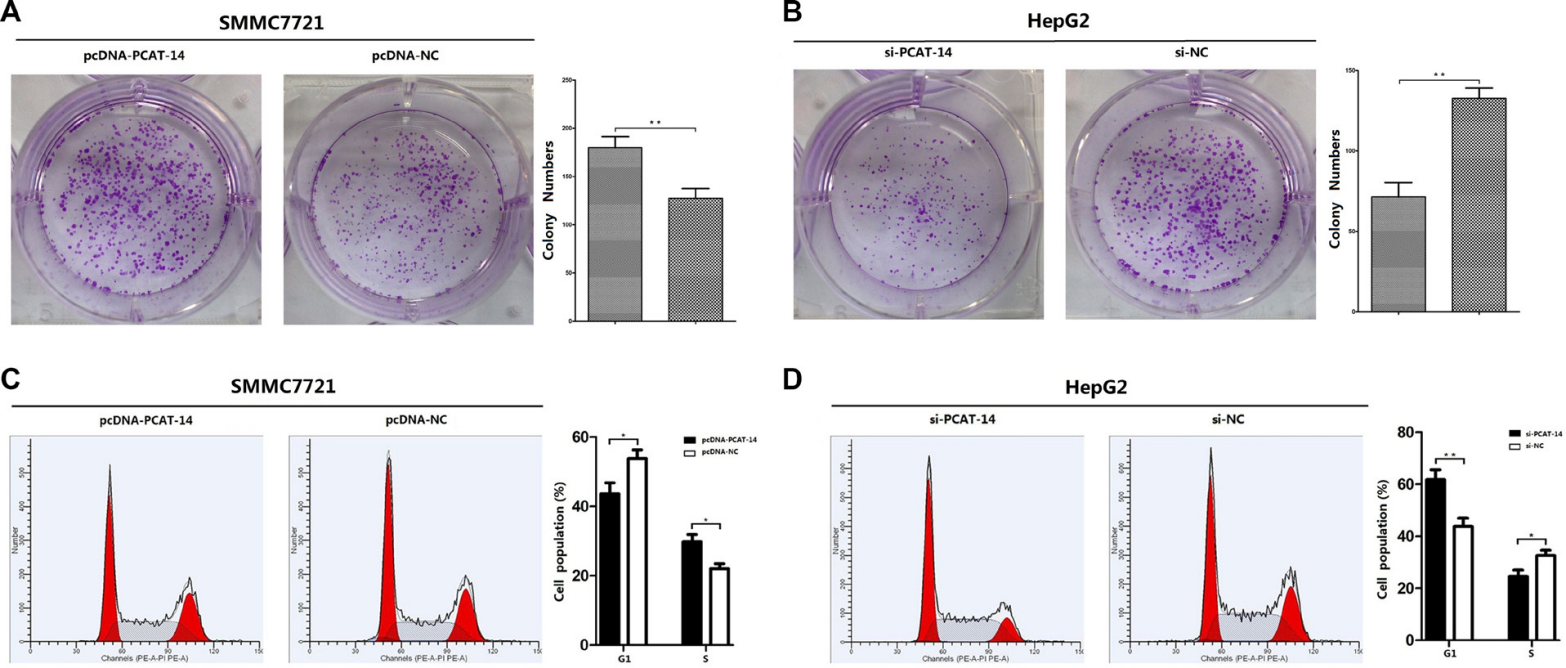
(A) Clonogenic assays were performed with SMMC7721 cells. The number of colonies formed by cells treated with pcDNA-PCAT-14 was more than that of pcDNA-NC-treated cells (*P* < 0.01); (**B**) Clonogenic assays were performed with HepG2 cells. The number of colonies formed by cells treated with si-PCAT-14 was fewer than that of si-NC-treated cells (*P* < 0.01); (**C**) In SMMC7721 cells, PCAT-14-overexpressed cells showed an decrease in the number of cells in G1 phase (40.18%) and S phase (27.44%) compared with the negative control (G1, 56.52%; S, 20.64%,*P* < 0.05); (**D**) In HepG2 cells, PCAT-14 lowexpression showed an increase in the number of cells in G1 phase (62.46%) and S phase (21.09%) compared with the negative control (G1, 43.42%; S, 29.92%, *P* < 0.01).

**Figure 4:**
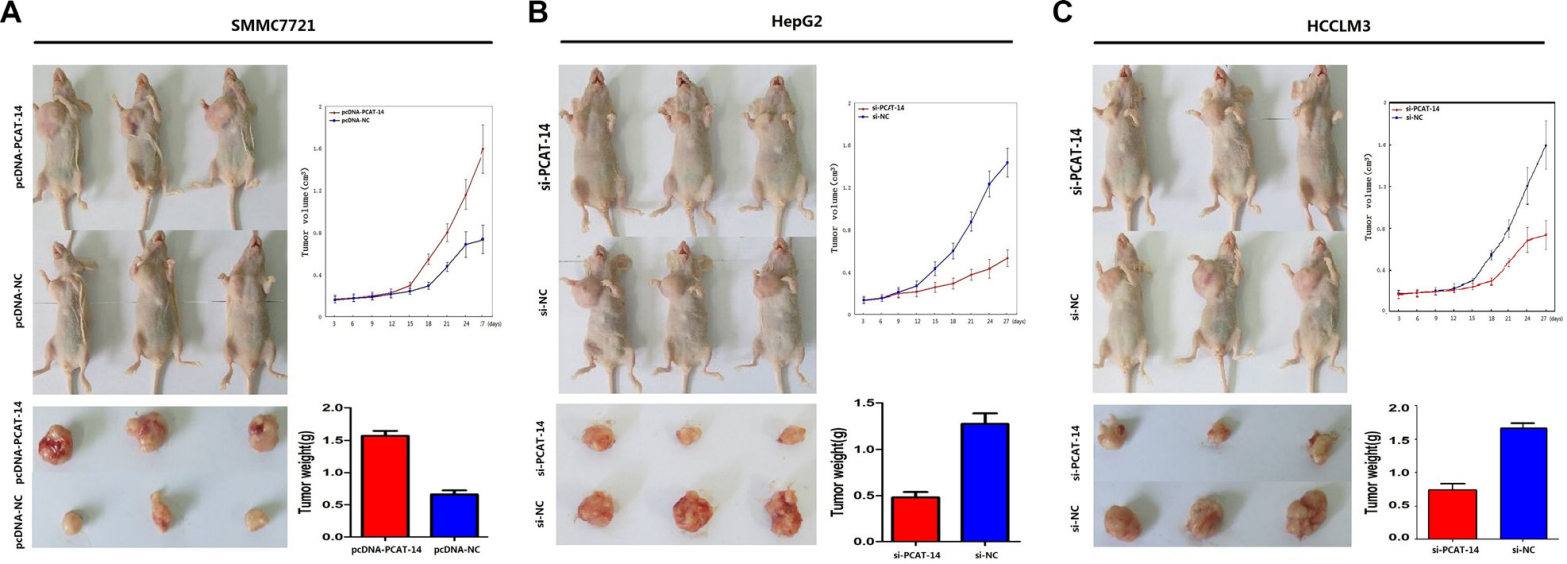
(A) Twenty-eight days after the mice were injected with SMMC7721 transfected by pcDNA-PCAT-14, the weight of the tumors were much higher than pcDNA-NC group (*P* < 0.01); (**B**) The weight of tumors in nude mice injected with HepG2 transfected by si-PCAT-14 were lower than si-NC group (*P* < 0.01); (**C**) The weight of tumors in nude mice injected with HCCLM3 transfected by si-PCAT-14 were lower than si-NC group (*P* < 0.01).

A significant change in proliferation rate was observed using the MTT assay 72 hours after transfection with pcDNA-PCAT-14 or si-PCAT-14 when compared to pcDNA-NC or si-NC in SMMC7721 and HepG2 cells (*P* < 0.01, Figure 6A, 6B). Consistent with the MTT assay, up-regulation of PCAT-14 in SMMC7721 cells increased the number and size of foci (pcDNA-PCAT-14 vs. pcDNA-NC: 184 ± 18 vs. 121 ± 14, *P* < 0.01, Figure 3A). In contrast, depletion of PCAT-14 in HepG2 cells decreased the number and size of foci (si-PCAT-14 vs. si-NC: 76 ± 14 vs. 133 ± 21, *P* < 0.01, Figure 3B). To test whether PCAT-14 could affect the tumorigenicity of HCC cells *in vivo*, a xenograft model of nude mice was used. Twenty-eight days after the mice were injected with SMMC7721 cells transfected with pcDNA-PCAT-14, the tumor weight was much higher in the cDNA-PCAT-14 group than in the control pcDNA-NC group (1.54 ± 0.23g vs. 0.68 ± 0.16g, *P* < 0.01, Figure 4A). The tumor weight in mice injected with HepG2 cells transfected with si-PCAT-14 was lower than in the control si-NC group (0.48 ± 0.12g vs. 1.29 ± 0.19g, *P* < 0.01, Figure 4B). The same phenomenons was observed in HCCLM3 (Figure 4C).

**Figure 5:**
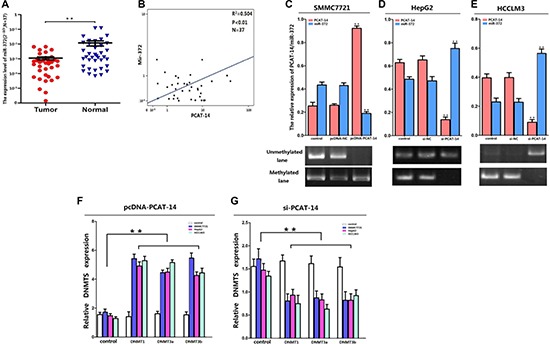
(A)The results of qRT-PCR showed miR-372 was lowexpressed (*P* < 0.01) in HCC tissue samples; (**B**) The expression levels of PCAT-14 and miR-372 were negative correlation by linear regression analysis accroading to the results of qRT-PCR in 37 HCC tissue samples (R^2^ = 0.504, *P* < 0.01); (**C**) The expression of miR-372 was significantly inhibited in SMMC7721 when PCAT-14 was overexpressed (*P* < 0.01) and MSP analysis showed PCAT-14 overexpression promoted the methylation of miR-372 CpG islands in SMMC7721 cells; (**D**, **E**) PCAT-14 silencing markedly promoted miR-372 expression (*P* < 0.01) and inhibited the CpG islands methylation in HepG2 and HCCLM3 cells; (**F**, **G**) The expression levels of DNMT1, DNMT3a, and DNMT3b were obviously increased when PCAT-14 was overexpressed (*P* < 0.01) and the contrary result was observed when PCAT-14 was knocked-down (*P* < 0.01).

**Figure 6:**
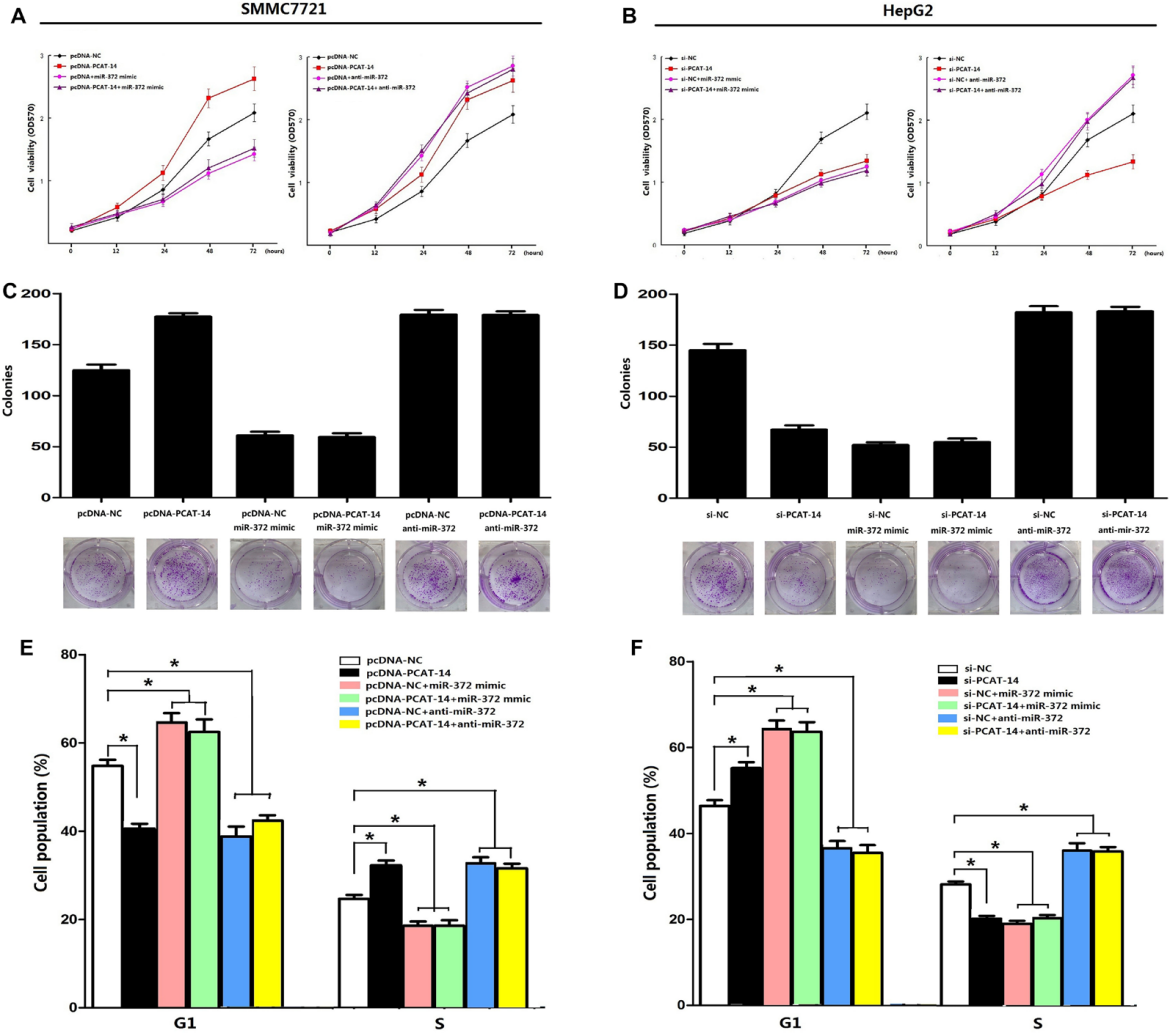
(A, B) A significant change in the proliferation rate was observed with the MTT assay 72 hours after transfection with pcDNA-PCAT-14 or si-PCAT-14 when compared to pcDNA-NC or si-NC in SMMC7721 or HepG2 cells respectively (*P* < 0.01) and miR-372 could block up the influence; (**C**, **D**) Clonal colony-forming assay showed miR-372 could eliminate the effect of PCAT-14 on colony-forming ability in SMMC7721 and HepG2 cells. (**E**, **F**) Flow cytometry analysis by PI staining indicated that miR-372 overexpression or lowexpression could eliminated the effect of PCAT-14 up-regulated or knockdown on G1/S phase in SMMC7721 or HepG2 cells.

### PCAT-14 regulates cancer cell cycle

The effect of PCAT-14 on the cell cycle was analyzed using flow cytometry analysis. In SMMC7721 cells, PCAT-14 overexpression decreased the number of cells in G1 phase (40.18%) and S phase (27.44%) compared with the negative control (G1, 56.52%; S, 20.64%, Figure 3C). In HepG2 cells, PCAT-14 downregulation increased the number of cells in G1 phase (62.46%) and S phase (21.09%) compared with the negative control (G1, 43.42%; S, 29.92%, Figure 3D). These results suggest that PCAT-14 could affect cell proliferation by regulating the G1/S phase.

### Expression of miR-372 negatively correlates with PCAT-14 expression in HCC

In this study, we used the same HCC samples for qRT-PCR and *in situ* hybridization as we have previously used for miR-372 analysis [22]. Analysis of HCC samples from 120 patients revealed a negative correlation between PCAT-14 and miR-372 (Figure 1A–1F). There was a significant negative association between PCAT-14 and miR-372 analyzed in the same specimens, using the Spearman Rank C test (Table 3, *P* < 0.01). Consistent with the results of *in situ* hybridization, miR-372 expression also negatively correlated with PCAT-14 expression by using linear regression analysis of 37 clinical HCC samples analyzed by qRT-PCR (R^2^ = 0.504, *P* < 0.01, Figure 5A–5B).

**Table 3:** Correlation analysis between PCAT-14 and miR-372 expression according to *in situ* hybridization in 120 patients with HCC

	PCAT-14 (0)	PCAT-14 (1+)	PCAT-14 (2+)	PCAT-14 (3+)	*P*
miR-372 (0)	1	2	13	17	**< 0.01**
miR-372 (1+)	1	3	30	20	−
miR-372 (2+)	3	7	3	3	−
miR-372 (3+)	4	8	4	1	−

Notes: Bold fonts indicate Statistically significant.

### PCAT-14 inhibits miR-372 expression in HCC by inducing methylation of CpG islands in miR-372 promoter

To investigate the relationship between PCAT-14 and miR-372, we evaluated the expression of miR-372 in three HCC cell lines (SMMC7721, HepG2, and HCCLM3) transfected with either pcDNA-PCAT-14 or si-PCAT-14. As shown in Figure 5C, the expression of miR-372 was inhibited in SMMC7721 cells when PCAT-14 was overexpressed (*P* < 0.01). In contrast, PCAT-14 silencing promoted miR-372 expression in HepG2 and HCCLM3 cells (Figure 5D, 5E, *P* < 0.01). We have previously demonstrated that aberrant DNA methylation of CpG islands upstream of the miR-372 promoter induces epigenetic silencing of miR-372 in HCC cells [22]. To explore the mechanisms of the negative miR-372 regulation by PCAT-14, we analyzed the levels of three active DNA methyltransferases (DNMT1, DNMT3a, and DNMT3b) in HCC cells by qRT-PCR when PCAT-14 was aberrantly expressed. The expression of DNMT1, DNMT3a, and DNMT3b was increased when PCAT-14 was overexpressed (Figure 5F, *P* < 0.01). An opposite result was observed when PCAT-14 was knocked-down (Figure 5G, *P* < 0.01), suggesting that PCAT-14 might regulate miR-372 methylation. In addition, MSP analysis showed that PCAT-14 overexpression promotes the methylation of miR-372 CpG islands in SMMC7721 cells, while PCAT-14 suppression inhibits the CpG islands methylation in HepG2 and HCCLM3 cells (Figure 5C–5E). Together, these findings indicate that in HCC, PCAT-14 inhibits miR-372 expression through inducing methylation of CpG islands in the miR-372 promoter.

### PCAT-14 regulates HCC cell proliferation and invasion depending on miR-372

Next, we investigated the miR-372 effect on PCAT-14 expression, proliferation, cell cycle, and invasion of HCC cells. SMMC7721 and HepG2 cells were transfected with pcDNA-NC, pcDNA-PCAT-14, pcDNA-NC+miR372 mimic, pcDNA-PCAT-14+miR-372mimic, pcDNA-NC+anti-miR-372, pcDNA+anti-miR-372 or si-NC, si-PCAT-14, si-NC+miR-372 mimic, si-PCAT-14+miR-372 mimic, si-NC+anti-miR-372, or si-PCAT-14+anti-miR-372. First, we assessed the proliferation of SMMC7721 and HepG2 cells using an MTT assay. We found that miR-372 mimic and anti-miR-372 could suppress the effect of pcDNA-PCAT-14 or si-PCAT-14 in SMMC7721 or HepG2 cells (Figure 6A, 6B). Similar results were obtained when the cell proliferation was analyzed by a clonal colony-forming assay (Figure 6C,6D). Cell cycle analysis using PI staining indicated that miR-372 overexpression or suppression could eliminate the effect of PCAT-14 on G1/S phase in SMMC7721 or HepG2 cells (Figure 6E, 6F). In addition, trans-well assay indicated that miR-372 could block the influence of PCAT-14 on cellular invasion and migration (Figure 2C, 2D). These results suggest that PCAT-14 regulates HCC cell proliferation, cycle, and invasion depending on miR-372.

### PCAT-14 regulates ATAD2 expression and activation of Hedgehog pathway via miR-372

**Figure 7:**
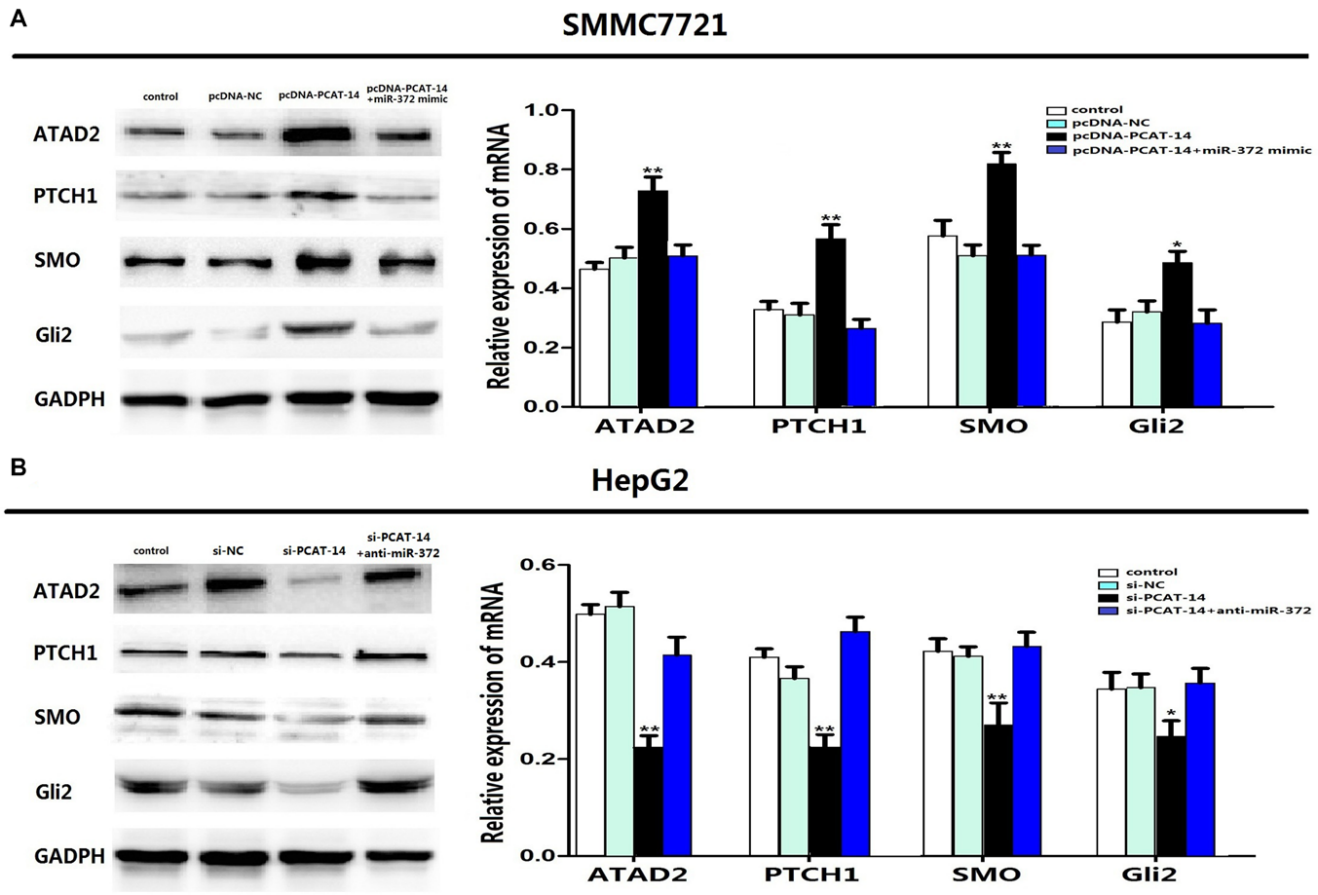
(A) Overexpression of PCAT-14 could up-regulated the expression of ATAD2, PTCH1, SMO, Gli2 in SMMC7721 cells and miR-372 mimic could eliminate the regulation. (**B**) In HepG2 cells, lowexpression of PCAT-14 could down-regulated the expression of ATAD2, PTCH1, SMO, Gli2 and anti-miR-372 could eliminate the regulation.

We have previously shown that ATAD2 is one of target genes of miR-372, and that ATAD2 regulates the Hedgehog pathway (Hh pathway) to influence HCC cell proliferation and metastasis [25, 26]. To investigate the role of PCAT-14 in the regulation of ATAD2 and the Hh pathway, we transfected pcDNA, pcDNA-PCAT-14, pcDNA-PCAT-14+miR-372 mimic or si-NC, si-PCAT-14, or si-PCAT-14+anti-miR-372 in SMMC7721 or HepG2 cells. Gene and protein levels of ATAD2 and Hh pathway key proteins (PTCH1, SMO, Gli2) were analyzed by qRT-PCR and western blotting, respectively. Overexpression of PCAT-14 increased the expression of ATAD2, PTCH1, SMO, and Gli2 in SMMC7721 cells, and miR-372 mimic reversed the effect. In HepG2 cells, suppression of PCAT-14 inhibited the expression of ATAD2, PTCH1, SMO, and Gli2, and anti-miR-372 eliminated the regulation (Figure 7A–7D). These findings indicate that in HCC cells, PCAT-14 regulates ATAD2 expression and Hh activation via miR-372.

## DISCUSSION

LncRNAs can regulate protein-coding genes, transcription, and post-transcription, and play important roles in biological processes [25, 26]. Many functions of lncRNAs in biological processes have been proposed, including cardiovascular diseases, inflammatory responses, and cancers [27, 28]. These roles are mediated by epigenetic regulation, and transcriptional and post-transcriptional regulation [29]. A number of lncRNAs have been identified in HCC, such as H19, HOTAIR, MALAT1, and MEG3 [30, 31].

PCAT-14 was first reported in prostate cancer. Down-regulation of PCAT-14 in prostate cancer has been associated with an increased probability of metastatic progression and mortality across multiple independent datasets and ethnicities [20]. In-vitro data confirmed that low PCAT-14 expression increases migration of prostate cancer cells, while overexpression of PCAT-14 reduces their growth, migration, and invasion [20, 21]. The above studies suggested that PCAT-14 suppresses development of prostate cancer. However, the function and regulation of PCAT-14 in HCC remained unknown.

In this study, we found a negative correlation between PCAT-14 and miR-372 in HCC tissues. We have previously shown that aberrantly high DNA methylation in the miR-372 gene promoter induces epigenetic silencing of miR-372 in HCC and that miR-372 inhibits cancer cell proliferation and invasion. Thus, we speculated that PCAT-14 might regulate the expression of miR-372, thus regulating HCC cell invasion and proliferation. It has been reported that lncRNAs could adjust the methylation status of target genes, such as LncRNA PVT1 regulates prostate cancer cell growth by inducing methylation of miR-146a, LncRNA DBCCR1–003 regulates methylation of DBCCR1 via DNMT1 in bladder cancer, and LncRNA RP5–833A20.1 induces methylation of NFIA in U251 cells [32–34]. To investigate the relationship between PCAT-14 and miR-372 in HCC, we evaluated the expression of miR-372 in three HCC cancer cell lines (SMMC7721, HepG2, and HCCLM3) transfected with either pcDNA-PCAT-14 or si-PCAT-14. Results showed that overexpression of PCAT-14 inhibited miR-372 expression and suppression of PCAT-14 elevated miR-372 expression. To explore the mechanisms of the negative regulation of miR-372 by PCAT-14, we analyzed the levels of three active DNA methyltransferases (DNMT1, DNMT3a, and DNMT3b) in HCC cells aberrantly expressing PCAT-14. The results demonstrated that PCAT-14 could up-regulate the expression of DNMT1, DNMT3a, and DNMT3b, suggesting that PCAT-14 might regulate miR-372 methylation. In addition, MSP analysis provided evidence that PCAT-14 overexpression promotes the methylation of miR-372 CpG islands in SMMC7721 cells, and PCAT-14 suppression inhibits the CpG islands methylation in HepG2 and HCCLM3 cells. Together, these findings indicate that in HCC cells, PCAT-14 inhibits the miR-372 expression through inducing the methylation of CpG islands in its promoter. Furthermore, our results show that miR-372 overexpression or downregulation can eliminate the effect of PCAT-14 on cell proliferation, invasion, and cell cycle in SMMC7721 and HepG2 cells, suggesting that PCAT-14 regulates the HCC carcinogenesis depending on miR-372.

The ATPase family AAA domain-containing protein 2, ATAD2, is highly expressed in several types of tumors, such as breast cancer, lung cancer, and large B-cell lymphoma [35–40]. ATAD2 is one of the target genes of miR-372, and regulates the Hh pathway to influence HCC cell proliferation and metastasis [23, 24]. To investigate the role of PCAT-14 in ATAD2 and Hh signaling, we altered the expression of PCAT-14 in SMMC7721 and HepG2 cells, and evaluated gene and protein levels of ATAD2 and Hh pathway key proteins (PTCH-1, SMO, and Gli2). Results showed that PCAT-14 could promote the expression of ATAD2, PTCH-1, SMO, and Gli2, while miR-372 could inhibit them, suggesting that PCAT-14 regulates the ATAD2 expression and Hedgehog pathway via miR-372.

**Figure 8:**
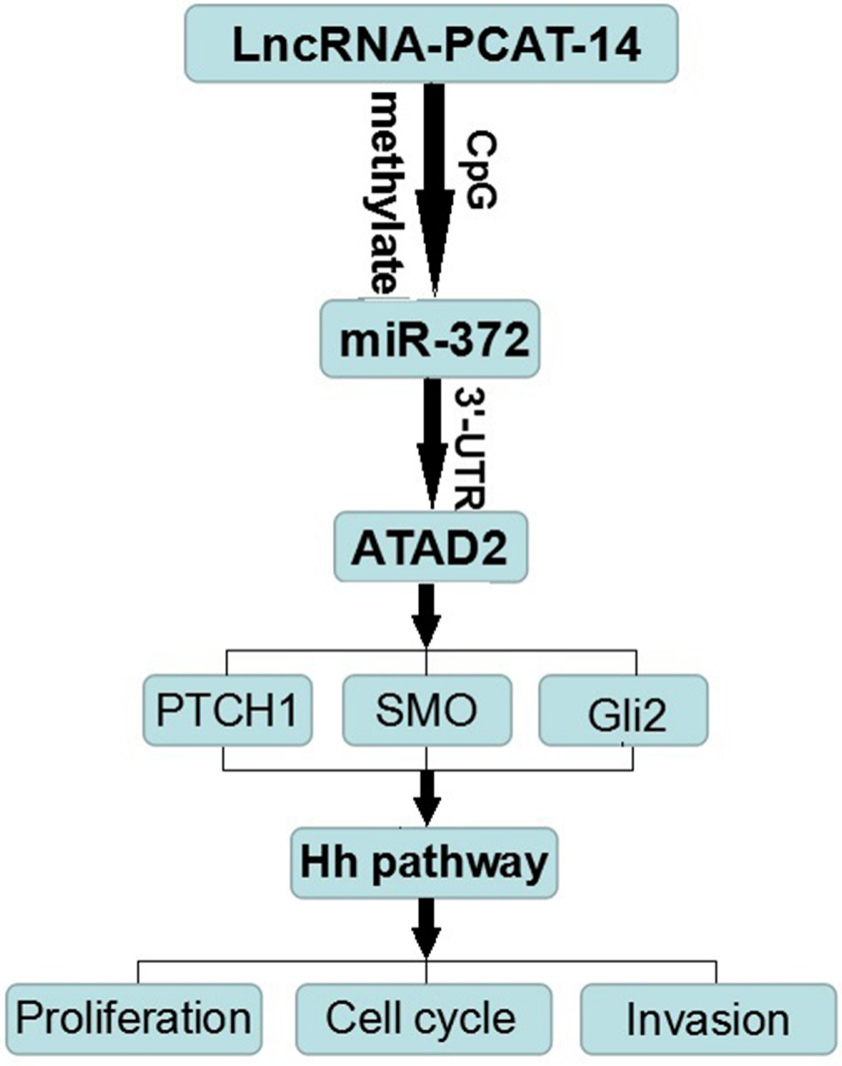
The molecular mechanism of PCAT-14 action in HCC carcinogenesis

In summary, here we demonstrate the function of PCAT-14 in HCC carcinogenesis by providing the following experimental evidence (Figure 8). 1) HCC tissues and cells express high levels of PCAT-14, which promotes HCC cell proliferation and invasion. 2) PCAT-14 inhibits miR-372 expression by inducing the methylation of CpG islands in the miR-372 promoter. 3) PCAT-14 regulates HCC cell growth and invasion depending on miR-372. 4) ATAD2 is one of the target genes of miR-372, and regulates the Hh pathway to influence HCC proliferation and metastasis. 5) PCAT-14 regulates ATAD2 expression and Hedgehog pathway via miR-372. Together, these findings indicate that PCAT-14 plays an important role in HCC carcinogenesis and may provide a new target for HCC detection and treatment. Future studies should determine the precise mechanisms of the aberrant expression of PCAT-14 in HCC.

## MATERIALS AND METHODS

### Patient tissue samples and liver cancer cell lines

HCC tissue slice samples were obtained from 120 patients (51 males and 69 females) diagnosed with HCC who had undergone a routine hepatic resection in the First Affiliated Hospital of China Medical University between January 2007 and January 2009 [22]. The follow-up period for sur*vivo*rs was 5 years. None of the patients had received preoperative radiotherapy or chemotherapy prior to surgical resection. A total of 37 paired fresh specimens, including both tumor tissues and the corresponding paired noncancerous parenchyma, were snap-frozen in liquid nitrogen and stored at −70°C immediately after resection until processing. Histological diagnosis and differentiation were evaluated independently by three pathologists according to the WHO classification system [41]. The project protocol was approved by the Institutional Ethics Committee of China Medical University prior to the initiation of the study. All patients provided written informed consent for the use of the tumor tissues for clinical research. The liver cancer cell lines Huh7, HCCLM3, HepG2, SMMC7721, PLC5, and QGY7701 and the normal liver cell line LO2 were obtained from Shanghai Cell Bank (Shanghai, China).

### RNA preparation and quantitative real-time PCR

Total RNA was extracted from approximately 100 mg of the 37 paired tissue samples and liver cancer cell lines using TRIzol reagent ( Invitrogen Company, USA) according to the manufacturer's instructions. The PCAT-14 primers were purchased from Takara Company (Japan). The GADPH gene was used as a reference control for PCAT-14. The relative gene expression was expressed as ΔCt = Ct gene - Ct reference and the fold change in gene expression was calculated using the 2^−ΔΔCt^ method [42].

### Western blot

Cells were washed with ice-cold PBS and lysed by RIPA (Beyotime, China) containing protease inhibitors (Beyotime, China). Protein lysates were separated in 10 % SDS-PAGE and transferred onto PVDF membrane (Millipore, USA). The membranes were blocked by 5 % skim milk in TBST buffer, and incubated with primary antibodies for ATAD2, PTCH1, SMO, and Gli2 (Abcam, UK) overnight at 4°C. PVDF membranes were washed in TBST and incubated with horseradish peroxidase-conjugated secondary antibodies (ProteinTech Group, USA). Antibody against GAPDH (Cell Signaling Technology, USA) was used as an internal control. Proteins were visualized using ECL Western blotting substrate (Pierce, USA).

### *In situ* hybridization

The *in situ* hybridization kit was purchased from Boster Company (Wuhan, China) and used according to the manufacturer's instructions. Briefly, the tissue slides were hybridized with 20 ul of 5′-digoxigenin (DIG) LNA-modified-PCAT-14–3p. Results were independently scored by two experienced pathologists. The scoring of positive tumor cells was as follows: 0 (0%), 1 (1–10%), 2 (11–50%), 3 (51–80%) and 4 (>80%). The staining intensity was visually scored as follows: 0 (negative), 1 (weak), 2 (moderate) and 3 (strong). The PCAT-14 expression score was calculated from the value of percent positivity score multiplied by the staining intensity score. This value ranged from 0 to 12, and the tumors were classified as follows: negative (−), score 0; lower expression (1+), score 1–4; moderate expression (2+), score 5–8; and strong expression (3+), score 9–12. *In situ* hybridization PCAT-14 staining was grouped into two categories: low expression (0/1+) and high expression (2+/3+).

### Cell transfection

SMMC-7721 cells were transfected with pcDNA-lincRNA-PCAT-14 for overexpressing PCAT-14, and HepG2 cells were transfected with si-lincRNA-PCAT-14 for suppression of PCAT-14 using DharmaFECT Transfection Reagent (GE Healthcare, Little Chalfont, Buckinghamshire, UK) according to manufacturer's instructions. The pcDNA-NC and si-NC acted as negative controls, respectively. The pcDNA-lincRNA-PCAT-14, pcDNA-NC, si-lincRNA-PCAT-14 and si-NC were synthesized by Life Technology (Beijing, China). miR-372 mimic or anti-miR-372 (both purchased from Shanghai Genepharma Company) were transfected using Lipofectamine^®^ RNAiMAX Reagent (Invitrogen, USA) according to the manufacturer's instructions.

### Cell cycle analysis

SMMC-7721 or HepG2 cells seeded at a density of 5 × 105 per well in 6-well plates were transfected with pcDNA-lincRNA-PCAT-14 or si-lincRNA-PCAT-14. After 48 h, cells were trypsinized, fixed with 70% ethanol at 4°C, and washed with PBS. RNase A (100 μL) was added, and the mixture was incubated in a 37°C water bath for 30 min. Next, 400 μL of PI staining solution was added and samples were incubated at 4°C in the dark for 30 min; a computer was then used to detect and record the red fluorescence upon excitation at a wavelength of 488 nm.

### MTT assay

After transfection, 5000 cells/well were seeded in 96-well plates in media containing 10 % FBS and incubated for 0, 24 h, 48 h, 72 h. On the indicated days, 3- (4,5)-dimethylthiahiazo (-z-y1)-3,5-di-phenytetrazoliumromide (MTT) (KyeGEN BioTECH, Nanjing, China) was added into each well according to the manufacturer's instructions, and the cells were incubated for 4 h at 37°C. The supernatants were then removed and 150 mL of DMSO (Sigma-Aldrich, Germany) was added to each well to dissolve the formazan crystals. Absorbance levels were measured at the wavelength of 490 nm using an automatic microplate reader (Gene, HK). The data derived from triplicate samples are presented as mean ± s.d.

### Colony formation assay

After transfection, 500 cells were counted and seeded in 6-well plates. The plates were incubated for 10 days, and the cells were fixed by 4 % paraformaldehyde and stained using 0.1 % crystal violet. Colonies were counted only if they included 50 cells at least. Triplicate independent experiments were performed.

### Cell invasion assay

SMMC-7721 or HepG2 cells were transfected with pcDNA-lincRNA-PCAT-14 or si-lincRNA-PCAT-14 for 48 h, and seeded onto a synthetic basement membrane in the inset of a 24-well culture plate. In the invasion assay, polycarbonate filters coated with 50 μL Matrigel (1:9, BD Bioscience) were placed in a Transwell chamber (Costar). After incubation, filters were fixed and stained with 0.1% crystal violet solution. Non-invading cells were removed using a cotton swab, and invading cells on the underside of the filter were counted with an inverted microscope.

### Tumorigenicity experiments in nude mice

Eighty male nude mice weighing 18 to 20 g, provided by Shanghai Laboratory Animal Center (Chinese Academy of Science, China), were bred under aseptic conditions; the animals were housed in an area with a constant humidity of 60–70% and a room temperature of 18–20°C. Animal maintenance, husbandry and experimental procedures were performed in accordance with the rules of China Medical University for the Use of Experimental Animals and approved by the Medical Animal Care and Use Committee of China Medical University (Shenyang, China). Mice were separated into four groups: pcDNA-lincRNA-PCAT-14 VS pcDNA-NC; si-lincRNA-PCAT-14 VS si-NC. The corresponding cell groups were administered by a subcutaneous injection (0.1 ml of a solution containing 1 × 10^4^ cells/ml). Mice were examined every 5 days, and sacrificed 28 days after the initial subcutaneous injection. The tumors were resected and weighed.
